# Extracellular vesicles as modulators of glioblastoma progression and tumor microenvironment

**DOI:** 10.3389/pore.2024.1611549

**Published:** 2024-02-06

**Authors:** Jie Dai, Yong Jiang, Haoyue Hu, Shuang Zhang, Yue Chen

**Affiliations:** ^1^ Department of Pathology, Sichuan Clinical Research Center for Cancer, Sichuan Cancer Hospital & Institute, Sichuan Cancer Center, Affiliated Cancer Hospital of University of Electronic Science and Technology of China, Chengdu, China; ^2^ Department of Neurosurgery, Sichuan Clinical Research Center for Cancer, Sichuan Cancer Hospital & Institute, Sichuan Cancer Center, Affiliated Cancer Hospital of University of Electronic Science and Technology of China, Chengdu, China; ^3^ Department of Medical Oncology, Sichuan Clinical Research Center for Cancer, Sichuan Cancer Hospital & Institute, Sichuan Cancer Center, Affiliated Cancer Hospital of University of Electronic Science and Technology of China, Chengdu, China; ^4^ Department of Pathology, Beijing Shijitan Hospital, Capital Medical University, Beijing, China

**Keywords:** extracellular vesicles, tumor microenvironment, glioblastoma, exosomes, microvesicles

## Abstract

Glioblastoma is the most aggressive brain tumor with extremely poor prognosis in adults. Routine treatments include surgery, chemotherapy, and radiotherapy; however, these may lead to rapid relapse and development of therapy-resistant tumor. Glioblastoma cells are known to communicate with macrophages, microglia, endothelial cells, astrocytes, and immune cells in the tumor microenvironment (TME) to promote tumor preservation. It was recently demonstrated that Glioblastoma-derived extracellular vesicles (EVs) participate in bidirectional intercellular communication in the TME. Apart from promoting glioblastoma cell proliferation, migration, and angiogenesis, EVs and their cargos (primarily proteins and miRNAs) can act as biomarkers for tumor diagnosis and prognosis. Furthermore, they can be used as therapeutic tools. In this review, the mechanisms of Glioblastoma-EVs biogenesis and intercellular communication with TME have been summarized. Moreover, there is discussion surrounding EVs as novel diagnostic structures and therapeutic tools for glioblastoma. Finally, unclear questions that require future investigation have been reviewed.

## Introduction

Gliomas are common central nervous system (CNS) tumors that are classified according to morphology and molecular characteristics, graded according to malignancy. The World Health Organization (WHO) has classified gliomas into the four subtypes of grades I-IV as per histopathological standards [1]. Glioblastoma is the most malignant form of glioma, with an incidence of 0.59–3.69 per 100,000 people per year worldwide. Glioblastoma is considered the most fatal brain tumor in adults owing to its poor prognosis, with a median survival of 14–16 months after diagnosis despite receiving standard therapy and care [2]. With the development of the CNS 5th WHO standard, molecular tests have been included the classification criteria [3]. In the latest 5th WHO brain tumor classification, the previous use of the term primary glioblastoma was re-characterized as grade IV astrocytoma (IDH-wildtype), whereas secondary glioblastoma was re-characterized as grade IV astrocytoma (IDH-mutant) [4].

At present, many therapies for glioblastoma have failed to improve outcomes, including surgical tumor resection, temozolomide (TMZ) chemotherapy and radiation therapy, and immunotherapeutic approaches, with <5% patients surviving past 5 years [5, 6]. Glioblastoma heterogeneity (intra- and intertumoral), resistance, and insufficient tumor microenvironment (TME) understanding are critical challenges influencing effective therapy development [7–9]. Therefore, specific mechanisms that affect treatment and prognosis as well as new therapies need to be identified and investigated.

The TME includes components from the tumor niche and extracellular components surrounding tumor cells. The TME generally comprises tumor cells, extracellular matrix (ECM), blood vessels, and tumor-infiltrating immune cells (monocytes, macrophages, neutrophils, T cells, and others) [10]. However, the glioblastoma TME displays particular heterogeneity and complexity, and, in addition to the abovementioned components, contains resident microglia (constituting 30%–50% of the cellular content), neurons, astrocytes, oligodendrocytes, Glioblastoma stem cells (GSCs), and endothelial and vascular pericytes [11].

The TME favors tumor growth, invasion, angiogenesis, and immunosuppression in glioblastoma, which benefits from the communication between glioblastoma cells and the surrounding non-tumor cells [12]. This process involves multiple modes of communication, including direct cell-to-cell contact, soluble factors (chemokines, cytokines, and growth factors), and extracellular vesicles (EVs) [13]. EVs are different cell-derived membranous structures surrounded by a phospholipid bilayer membrane that encapsulates various signaling molecules, such as dsDNA fragments, RNA variants, proteins, lipids, and metabolites [14]. EVs have been detected in different human CNS diseases, including Alzheimer’s disease, Parkinson’s disease, and brain tumors, making them an area of interest and ongoing field of research during the last few years [15]. Glioma derived-EVs have a profound effect on the activity and synchrony of neuronal networks, though the specific mechanism has not been addressed sufficiently [16]. Moreover, brain metastases-derived EVs (Br-EVs) trigger low-density lipoprotein aggregation, which accelerates Br-EVs uptake by monocytes (key components in the brain metastatic niche) [17]. Several studies have shown that EVs are a potential source of new biomarkers, relevant to novel targeted therapeutics, or drug delivery materials for many tumors [18].

This review explores the biological functions of EVs and the mechanistic interactions between glioblastoma cells and TME via EVs. In addition, the potential role of EVs in the antitumor effects against glioblastoma has been discussed and explored.

## Biogenesis and isolation methods of EVs

EVs can be classified into three subclasses based on their origin and/or size: exosomes (50–90 nm in diameter), microvesicles (100–1,000 nm in diameter), and apoptotic bodies (100–5,000 nm in diameter) [19, 20]. Exosomes, small vesicles of endocytic origin, were first proposed in 2012 as a protective envelope for viable blood microRNAs (miRNAs) [21]. By contrast, microvesicles, also known as ectosomes, are larger molecules that are produced from a cell via direct external budding, whereas apoptotic bodies are produced in the form of blebs during programmed cell death [21, 22]. The specific biogenesis mechanisms of these EVs, which are still being investigated, are different.

### Biogenesis of exosomes

Exosomes are formed via an invagination of the endosomal plasma membrane and first divided into three compartments: early, recycling, and late endosomes [23]. Late endosomes subsequently accumulate to form intraluminal vesicles (ILVs) contained in multivesicular bodies (MVBs), which are then secreted during the fusion of MVBs with the cell membrane of various cell types [24]. There are at least two known mechanisms involved in ILV and exosome production: the endosomal sorting complexes required for transport (ESCRT)-dependent and ESCRT-independent pathways [25]. The ESCRT-dependent machinery comprises the significant functions for MVBs biogenesis. The ESCRT pathway consists of five components: ESCRT-0, ESCRT-I, ESCRT-II, ESCRT-III, accessory protein ATPases, and vacuolar protein sorting-associated protein 4 (VSP4). Among these, ESCRT-0 is involved in ubiquitin-dependent clustering and ESCRT-I/II and ESCRT-III induce bud formation and vesicle abscission, respectively. ATPases and VSP4 regulate the dissociation and recycling of the ESCRT subunit [26]. In particular, ESCRT-0 binds to ILVs/MVBs, which are enriched in lipids (cholesterol and lysobisphosphatidic acid), and gathers ubiquitinated membrane proteins into these areas. Then, lipid-ubiquitinated domains begin to deform and invaginate, followed by the recruitment of ESCRT-I and ESCRT-II to stabilize and maintain the membrane status and prevent deformation. Subsequently, Vps20 and Snf7 (ESCRT-III subunits) are recruited into the invaginated lipid-ubiquitinated domain, which is deubiquitinated via polymerization to recruit Doa4. Finally, with the help of VSP4, vesicles are released from the limiting cell membrane along with the disassembling and recycling of the ESCRT-III complex [27]. Previous studies have shown that depletion of the ESCRT-0 proteins [Hrs, Tumor susceptibility 1 (TSG1)] and the ESCRT-l protein [Signal transducing adapter molecule 1 (STAM1)] reduced the secretion of exosomes [28]. However, one recent study suggested that the silencing of apoptosis-linked gene 2 interacting protein X (ALIX) could modify the protein composition of exosomes and reduce the release of exosomes, thereby demonstrating the significance of ALIX for exosome biogenesis [29].

**FIGURE 1:**
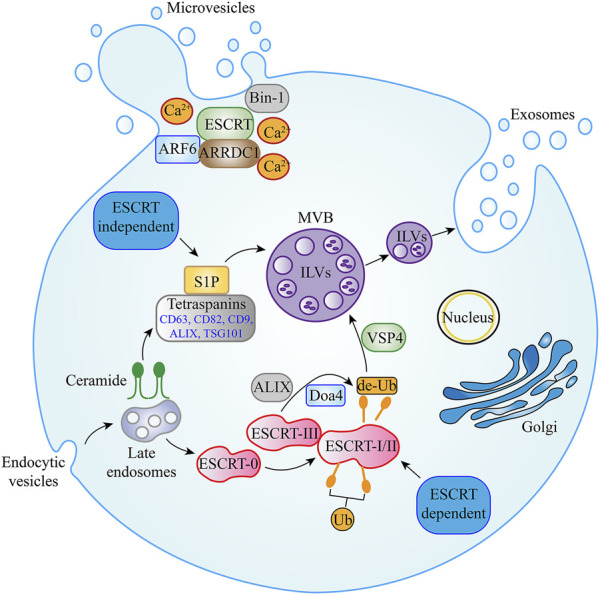
Biogenesis pathways of EVs. Multiple EV secretion mechanisms have been described. MVBs contain ILVs, which are formed during endosomal maturation. In ESCRT-dependent secretion, ESCRT-0, ESCRT-I, ESCRT-II, ESCRT-III, VSP4, Doa4, and ALIX proteins have been shown to promote exosome secretion. In exosome secretion via the ESCRT-independent pathway, ceramide, S1P, and tetraspanin family members such as CD63, CD82, CD9, ALIX, and TSG101 are essential. For plasma membrane-derived microvesicles, ARRDC1, ARF6, Bin-1, and Ca^2+^ in addition to some components of the ESCRT family (including ALIX and TSG101) have been shown to regulate outward budding. Abbreviation: EVs, Extracellular Vesicle; MVBs, Multivesicular bodies; ILVs, Intraluminal vesicles; ESCRT, endosomal sorting complexes required for transport; VSP4, Vacuolar protein sorting-associated protein 4; ALIX, Apoptosis-linked gene 2 interacting protein X; S1P, Sphingosine-1-phosphate; TSG101, Tumor susceptibility gene-101; ARRDC1, Arrestin domain-containing protein 1; ARF6, ADP-ribosylation factor 6.

Studies have found that some mammalian cells can form ILVs without abundant ESCRT components. With a deeper understanding, this approach is called the ESCRT-independent pathway, which operates to form MVBs and promote exosomal biogenesis through ceramide production. In this mechanism, ceramide can directly promote the membrane budding of ILVs or activate sphingosine-1-phosphate (S1P), subsequently binding to the MVB membrane receptors [30, 31]. By contrast, the tetraspanin family [such as CD63, CD82, CD9, ALIX, and the tumor susceptibility gene-101 (TSG101)] were consistently observed on the surface of exosomes, suggesting that these proteins constitute a separate mechanism for MVB and exosome biogenesis (Figure 1) [32].

### Biogenesis of microvesicles

In the past, it was difficult to distinguish microvesicles and exosomes because of their similar function. However, with the discovery of their biogenesis mechanisms, the two molecules were clearly differentiable owing to fundamental differences in their biogenesis mechanisms [33]. Microvesicles are produced directly from the outward budding of the plasma membrane where multiple mechanisms appear to be at play, although specific mechanisms are still being explored [34]. At present, the biogenesis pathway of arrestin domain-containing protein 1 (ARRDC1)-mediated microvesicles is one of the more recognized mechanisms. ARRDC1 is recruited to the plasma membrane along with elements of the ESCRT pathway generating microvesicles. This approach requires the involvement of VPS4 ATPase (an ESCRT pathway component), which participates in the ubiquitination of ARRDC1 [35]. In addition, ADP-ribosylation factor 6 and other ESCRT pathway components (including ALIX and TSG101) have recently been implicated in the regulation of the outward budding of microvesicles. Other microvesicles have been reported to utilize Bin-1 (ampiphysin) or Ca^2+^ to promote physical tension and molecular curvatures on the plasma membrane, thereby favoring microvesicle budding (Figure 1) [36, 37].

### Isolation methods of EVs

In order for microvesicles and exosomes to serve as viable tumor biomarkers, the EVs need to be isolated from the liquid biopsies. Traditional methods for exosome isolation are differential ultracentrifugation, density gradient centrifugation, ultrafiltration, immunoaffinity, polymer precipitation and size exclusion chromatography (SEC) [38]. Each method has advantages and disadvantages, of which ultracentrifugation has the advantages of being relatively cheap and mature, but it is time-consuming and is not convenient to operate, and may also damage exosomes [39]. Although density gradient centrifugation can obtain high purity EVs and avoid exosome damage, the operation is very complicated. In contrast, ultrafiltration is easy to operate, but may result in loss of exosomes of a small particle diameter [40]. The SEC method can balance the advantages of simple operation, economy, and maintaining biological function and structure, but it may lead to lipoprotein contamination [41]. Immunoaffinity has high specificity for exosome subtype isolation, but it is expensive. Polymer precipitation is a simple operation and suitable for large-volume samples, but there are potential contaminants [42]. The most widely used technique for microvesicle isolation is specific anticoagulants coupled with high-speed ultra-centrifugation. The use of particular anticoagulants is essential, which can preserve microvesicles counts (such as protease inhibitor anticoagulants) rather than diminish microvesicles counts (such as citrate and EDTA anticoagulants) [43]. Different methods should be used for EV isolation according to their physical properties or the surface protein types. In addition to the choice of isolation technology, it is also necessary to consider which liquids (serum, plasma, CSF) from which EVs are extracted, as well as considering the transportation, storage temperature, and storage time. Refer to the guidelines published by the International Society for Extracellular Vesicles for specific options [44].

## Glioblastoma-derived EVs specific cargos

EV biological functions are complex and diverse, and many pathways have not yet been completely understood. However, the key role of EVs is in cellular intercommunication, as mentioned earlier [45]. EVs can be released and used by normal and tumor cells, although the molecular composition of the two is different. Regardless of the physiological or pathophysiological state, the diverse cargo composition of EVs determines their function in various cellular processes. Tumor cells are known to produce more EVs compared with non-tumor cells and carry specific bioactive molecules (proteins and RNAs) [46]. Glioblastoma cells also release EVs, and the major cargos are presented in Table 1.

**TABLE 1:** List of RNAs isolated from Glioblastoma-EVs.

RNA type	Key findings	Reference
mRNAs	Glioblastoma-EVs mRNAs stimulate glioma cells proliferation and initiate angiogenesis in brain endothelial cells	[47]
miR-9-5p	Higher expressions of miR-9-5p and miR-138-5p are correlated to the shorter survival in astrocytoma grade IV, not in glioblastoma	[48]
miR-138-5p		
miR-25-3p	Glioblastoma-EVs microRNA miR-25-3p facilitates tumor proliferation and TMZ resistance by promoting C-MYC and cyclin E expression via FBXW7 downregulation	[49]
miR-27a-3p	Glioblastoma-EVs microRNA miR-27a-3p promotes cell proliferation and motility and promotes M2 macrophage polarization via the EZH1/KDM3A/CTGF axis	[50]
miR-30b-3p	Glioblastoma-EVs microRNA miR-30b-3p decreases cell apoptosis and increases proliferation by directly targeting RHOB	[51]
miR-182-5p	Glioblastoma-EVs microRNA miR-182-5p promotes tumor angiogenesis and proliferation via the targeting of KLF2 and KLF4	[52]
miR-1238	Glioblastoma-EVs microRNA miR-1238 facilitates TMZ resistance by directly targeting the CAV1/EGFR pathway	[53]
miR-1246	Glioblastoma-EVs microRNA miR-1246 accelerates cell proliferation and invasion and promotes M2 macrophage polarization by directly targeting the TERF2IP pathway	[54]
miR-124	Glioblastoma-EVs microRNA miR-124 suppresses the cell growth and inhibits M2 microglial polarization by regulating STAT3 signaling	[55]
miR-504	Glioblastoma-EVs microRNA miR-504 decreases tumor aggressiveness and inhibits M2 macrophage polarization by targeting Grb10 expression	[56]
miR-512-5p	Glioblastoma-EVs microRNA miR-512-5p promotes tumor progression and proliferation by targeting JAG1	[57]
lncRNA HOTAIR	Glioblastoma-EVs lncRNA HOTAIR promotes angiogenesis by induction of VEGFA expression	[58]
lncRNA SBF2-AS1	Glioblastoma-EVs lncRNA SBF2-AS1 is regulated by transcription factor ZEB1 and promotes TMZ resistance	[59]
lncRNA ROR1-AS1	Glioblastoma-EVs lncRNA ROR1-AS1 facilitates tumor progression via miR-4686 regulation	[60]
circKIF18A	Glioblastoma-EVs circKIF18A enhances the FOXC2 transcription factor activity, leading to increased angiogenesis	[61]
circSMARCA5	CircSMARCA5 and circHIPK3 were significantly less abundant in EVs from glioblastoma patients compared with unaffected controls, which suggests that these molecules can act as glioblastoma diagnostic biomarkers	[62]
circHIPK3		

Abbreviation: EVs, Extracellular Vesicle; miRNA, microRNA; lncRNA, long non-coding RNAs; TMZ, temozolomide; EZH1, Enhancer zeste homologue 1; KLF2/4, Kruppel-like factor 2/4; EGFR, epidermal growth factor receptor; TERF2IP, Telomeric repeat binding factor 2 interacting protein; STAT3, Signal transducer and activator of transcription 3; JAG1, Jagged 1; ZEB1, Zinc finger E-box binding homeobox 1.

### Proteins from Glioblastoma-EVs

There are many common EV surface protein biomarkers, including CD9, CD63, CD81, ALIX, TSG101, ESCRT proteins (Rab27a, Rab27b, and Rab11), and RNA-binding proteins. Likewise, there are many specific protein markers in Glioblastoma-EVs, including the anti-inflammatory enzymes CD39 and CD73 and the anti-inflammatory molecules programmed death ligand-1 (PD-L1) and indoleamine-2,3-dioxygenase 1, which are all involved in tumor progression [63]. Vascular endothelial growth factor (VEGF), matrix metalloproteinase 9 (MMP9), epidermal growth factor receptor (EGFR), and platelet-derived growth factor receptor, genes related to angiogenesis, are overexpressed in glioblastoma and presented on Glioblastoma-EVs surface [64]. Proteomic studies identified HSP27 and CD44, which participate in apoptosis inhibition and cell adhesion, respectively, as surface protein cargos in Glioblastoma-EVs [65].

### RNA types from Glioblastoma-EVs

Many key RNA types contained in Glioblastoma-EVs have been identified, including mRNAs, miRNAs, long non-coding RNAs (lncRNAs), and other rare RNAs. Indeed, >27,000 mRNA species have been identified in the serum of patients with glioblastoma, and 4700 of these mRNAs are specific to Glioblastoma-EVs. The biological processes of the 500 most abundant mRNA species in Glioblastoma-EVs are mainly distributed in the cellular process (27%), metabolic process (21%), biological regulation (10%), and developmental process (8%). Moreover, mRNAs belonging to angiogenesis, cell proliferation and migration, immune response, and histone modification functions have been identified [47]. In this study, the authors also found that the U87 glioma cells had increased 5-fold (incubated in normal growth medium) and 8-fold (incubated in medium with microvesicles) after 3 days of cultivation. And *in vitro* angiogenesis assay, there was a doubling of tubule length by the human brain microvascular endothelial cells within 16 h with the presence of microvesicles. These results revealed that glioblastoma-EVs can stimulate proliferation of the glioma cells and initiate angiogenesis in brain endothelial cells [47].

miR-9-5p, overexpressed in GSC-EVs, probably plays a distinct and complementary role to its angiogenic effects in endothelial cells (ECs) [66]. Higher expression levels of miR-9-5p and miR-138-5p are correlated to the shorter survival in glioblastoma patients carrying IDH mutation (now called astrocytoma grade IV), not in IDH wild type patients (glioblastoma) [48]. Moreover, the exosomal transfer of overexpressed miR-25-3p facilitated the proliferation and TMZ resistance of sensitive glioblastoma cells by promoting C-MYC and cyclin E expression via FBXW7 downregulation [49]. Glioblastoma-EV-packaged miR-27a-3p could promote M2 macrophage polarization via the EZH1/KDM3A/CTGF axis, and contribute to glioblastoma cell proliferation and motility, thereby increasing GSC tumorigenicity *in vivo* [50]. Glioblastoma-EV-delivered miR-30b-3p has been shown both *in vitro* and *in vivo* to decrease apoptosis and increase proliferation by directly targeting RHOB, offering a potential treatment strategy for glioblastoma [51]. Under hypoxic stress conditions, miR-182-5p was significantly upregulated in Glioblastoma-EVs, resulting in the promotion of tumor angiogenesis and tumor proliferation via the targeting of kruppel-like factor (KLF) 2 and KLF4(52). It was shown that high exosome-transferred miR-1238 level in TMZ-resistant glioblastoma cells could confer TMZ resistance by directly targeting the CAV1/EGFR pathway [53]. Qian et al. confirmed that exosomal miR-1246 in glioblastoma may play a role in M2 macrophage polarization to accelerate glioblastoma cell proliferation and invasion by directly targeting the telomeric repeat binding factor 2 interacting protein (TERF2IP) signaling pathway [54]. In addition, a set of underrepresented miRNAs have been identified in Glioblastoma-EVs. For example, a study demonstrated that miR-124 obtained from the U373MG glioblastoma cell line, in which an oncosuppressor is highly downregulated, exerted an antitumor effect by suppressing glioblastoma cell growth and inhibiting M2 microglial polarization by regulating the signal transducer and activator of transcription 3 (STAT3) signaling [55]. Moreover, miR-504 was identified as one of the most downregulated miRNAs in GSC-EVs that acted as a negative regulator of GSC migration, and miR-504 overexpression decreased tumorigenicity and induced microglia M1 phenotypes by targeting Grb10 expression, resulting in increased tumor aggressiveness and poor prognosis [56]. Besides the effect of miR-504, exosomal miR-512-5p, previously reported as an anti-oncogene in multiple solid tumors, was downregulated in glioblastoma. Furthermore, a reduction in miR-512-5p in glioblastoma played an important role in glioblastoma progression and proliferation by targeting Jagged 1 (JAG1) [57].

Aside from mRNAs and miRNAs, various specific lncRNAs have been identified in Glioblastoma-EVs. The lncRNA HOTAIR, an oncogene identified in gliomas, was involved in angiogenesis via its transmission into ECs through Glioblastoma-EVs, a process that requires VEGFA induction [58]. Zhang et al. found that high exosomal lncRNA SBF2-AS1 levels isolated from TMZ-resistant glioblastoma cells promoted TMZ resistance and were associated with poor prognosis. The SBF2-AS1 level was regulated by transcription factor zinc finger E-box binding homeobox 1 (ZEB1) and affected TMZ resistance in GBM cells [59]. Moreover, the exosome-packaged lncRNA ROR1-AS1 facilitated glioma progression via miR-4686 regulation, which was confirmed using a xenograft nude mice model [60].

Uncharacterized EV-isolated RNAs < 500 nucleotides were detected in circulating peripheral blood; however, their function could not be identified [67]. These RNAs, mapped in the intronic and intergenic regions, are also known as short non-coding RNAs and include piRNA, rRNA, snoRNA, snRNA, and yRNA [68]. Recently, circular RNAs (belonging to short non-coding RNAs) were considered a part of the specific RNA cargos of Glioblastoma-EVs. CircKIF18A can bind to the transcription factor FOXC2 to promote angiogenesis in glioblastoma by activating the PI3K/AKT signaling pathway [61]. CircSMARCA5 and circHIPK3 were significantly less abundant in Glioblastoma-EVs compared with unaffected controls, which suggests that these molecules can act as good glioblastoma diagnostic biomarkers when combined with the preoperative inflammatory markers of neutrophil to lymphocyte, platelet to lymphocyte, and lymphocyte to monocyte ratios [62]. Information concerning the major RNA cargos of Glioblastoma-EVs is summarized in Table 1.

## Glioblastoma-EVs and TME

**FIGURE 2:**
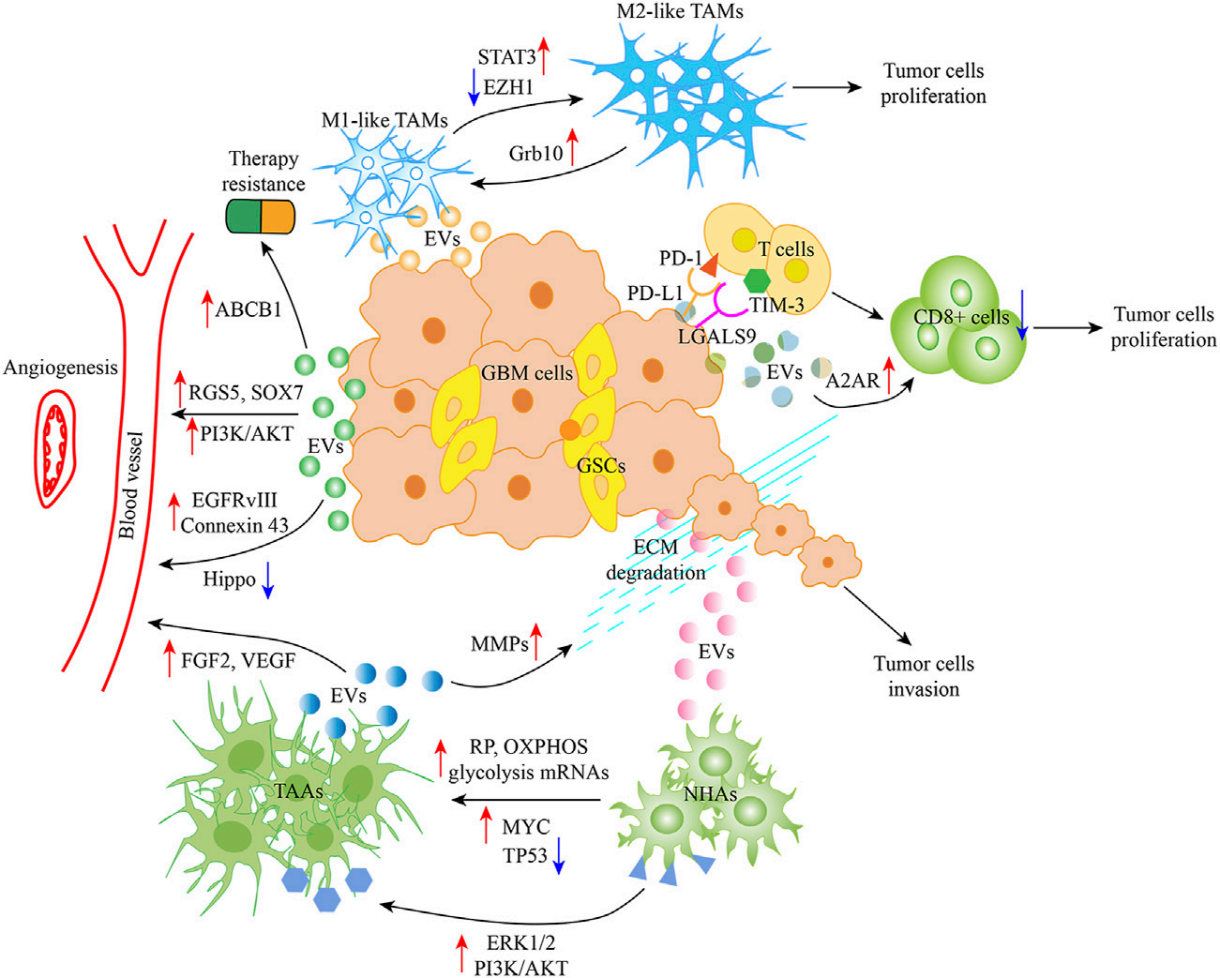
Glioblastoma-EVs and TME. Glioblastoma-EVs can induce TAMs with the M1 (antitumor) phenotype to the M2 (protumor) phenotype by activating the STAT3 signaling and targeting Grb10 as well as inhibiting EZH1. Glioblastoma-EVs can also increase PD-L1 and LGALS9 expression by interacting with the ligand PD-1 and TIM-3 present on activated T cell surface, thereby inhibiting T cell-associated immune response. Moreover, A2AR in T cells is involved in inhibiting T cell clonal proliferation. Meanwhile, Glioblastoma-EVs target ECs, which activate angiogenic mechanisms by regulating RGS5, SOX7, EGFRvIII, connexin 43, PI3K/AKT, and Hippo signaling. Moreover, ABCB1 appears to play a role in cancer therapy resistance, and Glioblastoma-EV uptake by NHAs leads to their conversion to TAAs, thereby favoring angiogenesis and tumor invasion by regulating FGF2 and VEGF and degrading ECM, respectively. Abbreviation: EVs, Extracellular Vesicle; TME, Tumor microenvironment; TAMs, Tumor-associated macrophages; STAT3, Signal transducer and activator of transcription 3; EZH1, Enhancer zeste homologue 1; PD-L1, Programmed death ligand-1; PD-1, Programmed cell death 1; TIM-3, T cell immunoglobulin domain and mucin domain-3; A2AR, Adenosine receptor 2A; RGS5, G protein signaling 5; EGFRvIII, EGFR mutant/variant III; ECs, endothelial cells; NHAs, Normal human astrocytes; TAAs, Tumor associated astrocytes; FGF2, Fibroblast growth factor 2; VEGF, Vascular endothelial growth factor; ECM, Extracellular matrix.

As discussed earlier, tumor cells, ECM, blood vessels, monocytes, macrophages, T cells, neurons, astrocytes, oligodendrocytes, ependymocytes, microglia, ECs, and pericytes are part of the glioblastoma TME. Important findings regarding the effect of Glioblastoma-EVs on different TME components, specifically on macrophages, microglia, vasculature, T cells, astrocytes, and glioblastoma cells, are discussed in this section (Figure 2).

### Effect on tumor-associated macrophages (TAMs)

Microglia, the resident brain innate immune cells previously called TAMs, together with monocytes and macrophages, play a key role in pathogen infection [69]. Macrophages are converted to either M1 (proinflammatory) or M2 (anti-inflammatory; promoting tumor progression) phenotype depending on TME cytokines and signaling molecules. Recent evidence has revealed that glioblastoma tissue supports the induction of such tumor-supportive phenotype modulation of TAMs through various EV-dependent mechanisms, as detailed below [70]. As noted above, several miRNAs (miR-27a-3p, miR-1246, miR-124, and miR-504) participate in microglial phenotype transformation. A 2022 study revealed that Glioblastoma-EV-derived miR-27a-3p could modify TAM phenotype *in vitro*, changing it to an M2 anti-inflammatory phenotype and promoting tumor progression by inhibiting enhancer zeste homologue 1 [50]. Qian et al. (2020) demonstrated that miRNA-1246 contained in Glioblastoma-EVs was transferred to recipient TAMs, resulting in an M2-like anti-inflammatory phenotype polarization of the macrophages. One of these targets is the TERF2IP, a transcription factor and regulator of telomere function, which activates the signal transducer and activator of transcription 3 signaling pathway and inhibits the NF-κB signaling pathway [54]. miRNA-124, which acts as an oncosuppressor, reduces cell growth and inhibits glycolytic pathways in colorectal cancer and breast cancer [71]. miR-124 present in microglia-derived small EVs was one of the most downregulated miRNAs in glioblastoma and may be associated with significantly reduced glioblastoma aggressiveness. However, macrophage-produced EVs could modify the macrophage antitumor phenotype to tumor-supportive phenotype when exposed to Glioblastoma-EVs. Moreover, the expression level of the M1 microglial polarization marker interleukin-6 was upregulated, whereas those of the M2 microglial polarization markers transforming growth factor-β and arginase 1 were significantly downregulated [55, 72]. Bier et al. showed that miR-504 contained in GSC-secreted EVs was transferred from GSCs to the surrounding neighboring cells such as microglia, subsequently promoting oncogenic functions. However, miR-504 was downregulated in glioblastoma cells and GSCs, suggesting that miR-504 is a negative regulator of glioblastoma tumorigenicity. Moreover, the overexpression of miR-504 in microglial cells upregulated the level of M1 phenotypic markers CD86 and tumor necrosis factor-α, indicating an increase in M1-like TAMs. These phenomena suggest that GSC-EVs induce microglial phenotype change. It has been speculated that the mechanism of this induction is related to Grb10 targeting [56]. In addition, Glioblastoma-EVs could promote tumor migration and invasion by accelerating ECM degradation in TME through macrophage phagocytic activity enhancement [73].

### Effect on ECs

The modification of angiogenesis in TME is a key mechanism that promotes glioblastoma growth via Glioblastoma-EVs. One mechanism through which glioblastoma cells accomplish this is through the transfer of miR-9-5p from GSC-EVs to brain ECs, which is associated with angiogenesis via the regulation of downstream G protein signaling 5 (RGS5), SOX7, and ABCB1(66). RGS5 and SOX7 have been identified to play critical roles in vascular growth by recruiting host pericytes and acting as the positive feedback regulators of VEGF signaling, respectively, thereby promoting physiological angiogenesis [74, 75]. Meanwhile, ABCB1 appears to play a role in cancer therapy resistance [76]. Ma et al. demonstrated that lncRNA HOTAIR delivered via the glioblastoma cell line A172 cell-derived EVs are involved in the promotion of angiogenesis via the upregulation of the expression of VEGFA, a well-known proangiogenic factor [58]. Another type of circular RNA, the microglia-derived exosomal circKIF18A, plays a role in proangiogenic effects, contributing in the RNA-aided nuclear entry of FOXC2, leading to its direct binding to the promoter of ITGB3 (C-X-C chemokine receptor type 4 and DLL4, and activating the PI3K/AKT signaling axis [61]. VEGF-C, a specific 120-kDa isoform of VEGF, transported via Glioblastoma-EVs (exosomes) have been identified in the promotion of EC viability, migration, and tubulation. The proangiogenic effects of VEGF-C have been attributed to inhibiting Hippo signaling to stimulate tafazzin expression in ECs by binding to VEGF receptor 2 [77]. The Glioblastoma-EV-mediated release of EGFR mutant/variant III (EGFRvIII) in the context of CD9 tetraspanin may enhance glioblastoma progression, including cell invasion and angiogenesis. Research has shown that EGFRvIII expression resulted in the downregulation of EV markers, notably including CD81 and CD82 [78]. Under 3% oxygen (hypoxia), the level of connexin 43 (Cx43) in exosomes secreted in the glioblastoma cell line U251 cells was elevated. However, compared with PBS (control), exosomal Cx43 remarkably promoted tube formation in human umbilical vein endothelial cells, thereby contributing to angiogenesis in hypoxic glioblastoma TME [79].

### Effect on T cells

Infiltrating T cells in the TME can be the target of Glioblastoma-EV-released cargos, various T cell types and mechanisms are involved in their interaction with the cargos [80]. Some findings have demonstrated that Glioblastoma-derived EVs can induce the formation of immunosuppressive monocytes such as non-classical monocytes (NCMs) and myeloid-derived suppressor cells (MDSCs) as well as increase PD-L1 expression [by binding with programmed cell death 1 (PD-1) located on the surface of activated T cells]. The formation of NCMs was PD-L1 dependent and that of MDSCs was PD-L1 independent. Further experiments revealed that PD-1 was present on NCM surfaces. NCMs and MDSCs interacted with T cells via PD-L1/PD-1 and inhibited T cell proliferation *in vitro*, thereby accelerating tumor formation and progression [81]. In low oxygen conditions (hypoxia), CD73 can be induced by the enzyme ecto-5′-nucleotidase, playing a crucial role in suppressing the systemic immune system and supporting tumor progression [82]. A recent study has suggested that CD73 is highly expressed in T cells and that this expression originates from Glioblastoma-EVs. Researchers also observed that elevated CD73 blocked the clonal proliferation of T cells by increasing adenosine concentration around T cells *in vitro*. This may be attributed to the production of adenosine requiring AMP degradation and aerobic glycolysis inhibition by activating adenosine receptor 2A in T cells, thereby starving the energy needed for T cell clonal proliferation [83]. LGALS9 (galectin-9) acts as the ligand for the T cell immunoglobulin domain and mucin domain-3 on CD4^+^ T cell surface, thereby leading to T cell apoptosis and poor prognosis in glioblastoma [84]. In other work, the percentages of neutrophils and CD8 + T cells in the cerebrospinal fluid (CSF) of patients with glioblastoma were reduced compared with that of healthy controls. LGALS9, a specific protein cargo contained in Glioblastoma-CSF-EVs, was shown to inhibit the antigen presentation of dendritic cells and cytotoxic T cell (CD8^+^) immunity, promoting tumor progression [85].

### Effect on astrocytes

Among the cell types associated with glioblastoma TME, astrocytes are the most likely to establish direct contact with glioblastoma cells. Astrocytes are directly involved in the formation of the primary blood–brain barrier (BBB) structure by closely associating with ECs and pericytes. In addition, astrocytes participate in modulating the diffusion of neurotransmitters across brain EVs [86]. A group of activated astrocytes, known as tumor-associated astrocytes, has been shown to be directly regulated by glioblastoma cells to enhance tumor growth and invasion and chemotherapy resistance. Some contents of astrocyte-derived EVs, such as HSP70, fibroblast growth factor 2, VEGF, and MMPs, have all been shown to be involved in neuroprotection, ECM remodeling, and angiogenesis, thereby supporting tumor processes [87]. Glioblastoma-EVs can regulate TME to benefit tumor survival by driving astrocytes toward a tumorigenic phenotype. In a study, normal human astrocyte molecules were modified to resemble known tumor signaling pathways (ERK1/2, PI3K, and AKT) to enhance their growth and migratory capacity in a semisolid matrix [88]. In a later study, Zeng et al. reported increased mitochondrial respiration and glycolysis in pretransformed astrocytes cultured with Glioblastoma-derived EVs owing to the direct transfer of ribosomal protein, OXPHOS, and glycolysis mRNAs. Indeed, patients with glioblastoma and high glycolysis enrichment scores had worse overall survival [89]. Results from a previous study suggest that reduced TP53 levels are related to an ECM modulation composition that favors tumor malignancy [90]. MYC, a proto-oncogene, is involved in cell cycle regulation, apoptosis, and GSC acquisition and maintenance [91]. Of note, MYC induction and decreased TP53 levels in Glioblastoma-EVs stimulated normal astrocytes to shift to a senescence-associated secretory phenotype (a tumor-supportive phenotype) to induce a favorable TME for tumorigenic abilities (growth and invasion) [92]. These findings suggest that Glioblastoma-derived EVs stimulate astrocytes to promote glioblastoma progression and invasion.

## Clinical applications of Glioblastoma-EVs

The diagnosis or post-treatment monitoring of glioblastoma is heavily dependent on imaging (e.g., magnetic resonance imaging [MRI]) and tissue biopsies. However, because the tumor mass can only be detected in MRI when it is sufficiently large, it is easy to miss the correct diagnosis and delay disease treatment. By contrast, it is impossible to obtain a real map of intratumoral heterogeneity using imaging, which limits the ability to predict and monitor treatment response. Tumor biopsies, particularly of brain tissues, are likewise not an ideal option owing to their invasiveness, which can cause brain swelling and hemorrhage [93]. In recent years, in an effort to improve the diagnostic sensitivity and outcome of glioblastoma, several non-invasive or minimally invasive strategies have been explored to optimize its diagnosis and monitoring including speed, cost, and patient acceptability. One of these strategies is the analysis of non-invasive liquid biopsy using Glioblastoma-derived EVs from breast milk, plasma, CSF, urine, and saliva, among other physiological fluids [94]. This new strategy showed significant advantages in terms of precise personalized diagnosis [95]. Glioblastoma-EVs carry numerous specific molecules that may be associated with oncogenesis and are released outside the cells. These circulating EVs are considered significant biomarker sources that may help improve diagnosis, monitoring, and follow-up [96]. The term “vesiclemia” defines EV concentration in the plasma, which can change according to the state of the patient. For example, vesiclemia is higher in patients with glioblastoma than in healthy donors. Moreover, vesiclemia is increased after recurrence. However, it has been shown to decrease after resective surgery therapy [97]. In line with this idea, several mRNAs, miRNAs, lncRNAs, circular RNAs, and proteins in plasmatic EVs have been shown to be upregulated or downregulated in patients with glioblastoma patients compared with healthy donors, as described above. Johan et al. found that 14 of the 30 tumor samples (47%) contained the EGFRvIII transcript, however, EGFRvIII was not found in serum exosomes of 30 normal control individuals, which indicated that EV RNA could be used as biomarkers for glioblastoma [47]. In addition, Johnny et al. found that the level of CSF EV miR-21 was higher than that in non-oncologic patients. Interestingly, only CSF EV miR-21 was suitable for disease diagnosis while no significant difference in miR-21 level was found in serum, suggesting that CSF EV miR-21 could be a feasible biomarker for the presence of glioblastoma [98]. These results suggest that EVs are a promising source of biomarkers during early diagnosis [95]. TMZ chemotherapy is commonly administered together with radiation therapy offered in glioblastoma treatment after surgical resection. As described earlier, some EV-derived proteins and RNA types confer radiotherapy or chemotherapy resistance and promote tumor recurrence. MiR-25-3p is overexpressed in EVs of TMZ resistant glioma cells and high miR-25-3p level in the serum of a glioblastoma patient is relevant to TMZ resistance and greater tumor size [49]. MiR-1238 level was significantly higher in serum exosomes of patients with recurrent glioblastoma compared to patients with primary glioblastoma [53]. Furthermore, a high level of lncRNA SBF2-AS1 in serum exosomes was associated with poor response to TMZ treatment in glioblastoma patients [59]. Therefore, the analysis of different cargos as well as the concentration of EVs isolated from liquid biopsy (plasma and CSF) are believed to represent predictive and prognostic biomarkers for glioblastoma therapy and recurrence [99].

EVs are a potential therapeutic tool for glioblastoma. EV formation promotes tumorigenesis and progression. Therefore, some researchers have expressed interest in exploring strategies to block EV formation, thereby inhibiting tumor development. Microvesicle biogenesis is modulated by lipid composition, cytoskeleton proteins, and Ca^2+^, all of which can alter membrane fluidity and deformability. Calpain comprises a family of cysteine proteases whose regulatory subunits contain a calcium-binding site. This cysteine protease family can apparently promote microvesicle shedding by remodeling the cytoskeleton. It has been reported that calpain inhibitors can reduce microvesicle production [100]. A low concentration (10–20 μg/mL) of calpeptin, the most widely used calpain inhibitor, has been reported to reduce microvesicle shedding from activated platelets [101]. In a subsequent study, Atanassoff et al. showed that 60 μM calpeptin reduced microvesicle release from human embryonic kidney 293 cells [102]. Researchers have also focused on the therapeutic effect of calpeptin on solid tumors. Experiments in a prostate cancer cell line (PC3) model and preclinical mouse model have demonstrated that calpeptin combined with docetaxel or methotrexate decreased intratumoral vascularisation and tumor proliferation [103]. However, a recent study found that calpeptin treatment increased the resistance of glioblastoma cells to TMZ chemotherapy, contradicting previous research [104]. Thus, the therapeutic effect of calpeptin in glioblastoma needs further exploration. Stillger et al. revealed that the overexpression of calpain-2 (member of calpain) and the calpain small subunit in glioblastoma contributed to TMZ resistance. A combination of the synthetic calpain inhibitor PD150606 and TMZ led to a decreased viability of U251N cells compared with TMZ treatment alone [105].

**FIGURE 3:**
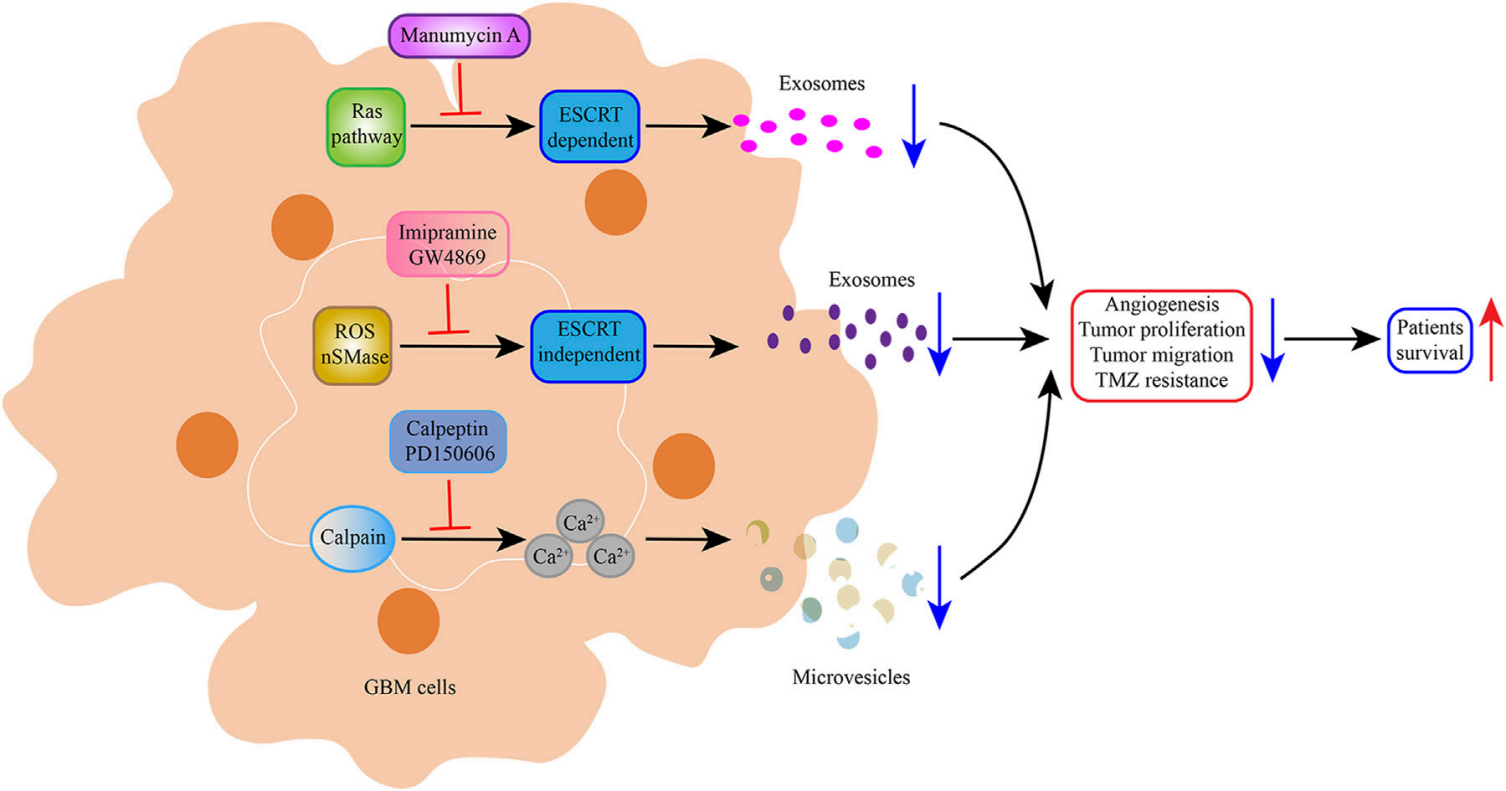
Schematic representation of the inhibition of Glioblastoma-EVs biogenesis and secretion. Manumycin A, imipramine, and GW4869 reduced exosome biogenesis and secretion, thereby prolonging the survival of patients with glioblastoma. Calpeptin and PD150606, potent calpain inhibitors, prevented the formation and budding of microvesicles, which decreased glioblastoma cell viability.

Exosome biogenesis is modulated by the ESCRT-dependent or -independent pathway. Manumycin A was identified as an inhibitor of exosome biogenesis in prostate cancer cells via the attenuation of the ESCRT-0 protein Hrs and ESCRT-accessory protein ALIX by Ras signaling inhibition [106]. An early report on the small molecule imipramine, an inhibitor of exosome biogenesis and secretion, highlighted the ability of combination chemotherapy (imipramine plus liposomal doxorubicin) to prolong the survival of patients with glioblastoma. This mechanism may be related to the inhibition of NADPH reactive oxygen species generation and conditional actin regulatory elements [107]. Neutral sphingomyelinase (nSMase) generates the bioactive lipid ceramide, which plays a key role in ESCRT-independent exosome generation. GW4869 has been identified as a specific non-competitive inhibitor of nSMase that reduced exosome release. Xu et al. performed animal experiments along with primary glioma cells G15-Luc-mimic cells. GW4869-treated mice displayed significantly reduced tumor size and exhibited better survival than controls (Figure 3) [108]. EVs are less immunogenic than standard transfection agents and can pass through BBB to a certain degree, suggesting that they offer an effective mode of drug delivery to the target site. Some research groups have evaluated the role of anti-miR-9 in reversing multidrug resistance to TMZ and suppressing glioblastoma malignant phenotypes [109, 110].

## Conclusion and future perspectives

Glioblastoma is a devastating disease with a high mortality rate. Thus, new therapies are required to improve its prognosis. The chemotherapy resistance of tumor cells is a frequent obstacle to therapy. The deepening of knowledge about intercellular communication, discovery of EVs, and new biomarkers have enhanced the efficacy of therapies [111]. The discovery of EV structure, specific cargo components, and their potential clinical applications in various tumors are topics that are currently the focus of many studies. The study of the role of EVs in the glioblastoma TME has also emerged in the last few decades. EVs are involved in different ways in glioblastoma tumor cell proliferation, migration, and drug resistance. This review summarized the current understanding on Glioblastoma-EVs and their potential as diagnostic, prognostic, and therapeutic tools.

Various EV-derived cargos (proteins, mRNAs, miRNAs, lncRNAs, and circular RNAs) act in glioblastoma target cells via intercellular communication and their ability to cross BBB. These findings suggest that they can be used as diagnostic and therapeutic tools [112].

However, many challenges regarding EV utilization in glioblastoma remain, including reaching a consensus regarding protocols for EV purification and criteria for characterization [113]. Thus, it is necessary to optimize EV isolation protocols before clinical translation can be considered. Moreover, the obtention and storage of EVs, especially exosomes, are challenging [112]. The use of innovative strategies to enhance exosome storage and long-term stability are emerging to preserve the physicochemical and biological properties of EVs, which may be crucial for clinical application. Taken together, although huge advances have been made in understanding the roles of EVs in glioblastoma, overcoming the aforementioned barriers may help EVs become essential components in the routine treatment of glioblastoma in the future.

## Figures and Tables

**Table:** 

**ALIX**	Apoptosis-linked gene 2 interacting protein X
**ARRDC1**	Arrestin domain-containing protein 1
**ARF6**	ADP-ribosylation factor 6
**ARG1**	Arginase 1
**A2AR**	Adenosine receptor 2A
**BBB**	Brain blood barrier
**CSF**	Cerebrospinal fluid
**CXCR4**	C-X-C chemokine receptor type 4
**Cx43**	Connexin 43
**CNS**	Central nervous system
**DCs**	Dendritic cells
**EVs**	Extracellular vesicles
**ECM**	Extracellular matrix
**ECs**	Endothelial cells
**EZH1**	Enhancer zeste homologue 1
**ESCRT**	Endosomal sorting complexes required for transport
**EGFR**	Epidermal growth factor receptor
**EGFRvIII**	EGFR mutant/variant III
**GSCs**	Glioblastoma stem cells
**ILVs**	Intraluminal vesicles
**IL-6**	Interleukin-6
**IDO1**	Indoleamine-2,3-dioxygenase 1
**JAG1**	Jagged 1
**KLF2/4**	Kruppel-like factor 2/4
**LBPA**	Lysobisphosphatidic acid
**LMR**	Lymphocyte to monocyte
**lncRNAs**	Long non-coding RNAs
**MVBs**	Multivesicular bodies
**MMP9**	Matrix metalloproteinase 9
**MA**	Manumycin A
**MDSCs**	Myeloid-derived suppressor cells
**miRNAs**	microRNAs
**NLR**	Neutrophil to lymphocyte
**NT5E**	Enzyme ecto-5′-nucleotidase
**NHAs**	Normal human astrocytes
**nSMase**	Neutral sphingomyelinase
**NCMs**	Nonclassical monocytes
**PDGFR**	Platelet derived growth factor receptor
**PD-L1**	Programmed death ligand-1
**PD-1**	Programmed cell death 1
**PM**	Plasma membrane
**PLR**	Platelet to lymphocyte
**ROS**	Reactive oxygen species
**RBPs**	RNA binding proteins
**RGS5**	G protein signaling 5
**RP**	Ribosomal protein
**SASP**	Senescence-associated secretory phenotype
**S1P**	Sphingosine-1-phosphate
**STAT3**	Signal transducer and activator of transcription 3
**TME**	Tumor microenvironment
**TSG101**	Tumor susceptibility gene-101
**TMZ**	Temozolomide
**TC**	Tumor cells
**TAMs**	Tumor-associated macrophages
**TGF-β**	Transforming growth factor-β
**TNF-α**	Tumor necrosis factor-α
**TERF2IP**	Telomeric repeat binding factor 2 interacting protein
**TIM-3**	T cell immunoglobulin domain and mucin domain-3
**TAAs**	Tumor associated astrocytes
**VEGF**	Vascular endothelial growth factor
**VSP4**	Vacuolar protein sorting-associated protein 4
**WHO**	World Health Organization
**ZEB1**	Zinc finger E-box binding homeobox 1
